# Clinically meaningful benefit: real world use compared against the American and European guidelines

**DOI:** 10.1038/s41408-017-0009-8

**Published:** 2017-12-14

**Authors:** Jessica J. Dreicer, Sham Mailankody, Farhad Fakhrejahani, Vinay Prasad

**Affiliations:** 1 Department of Medicine/OHSU, Portland, OR 97239 USA; 2 Myeloma Service/Memorial Sloan Kettering Cancer Center, New York City, NY USA; 3 National Cancer Institute/National Institutes of Health, New York City, NY USA; 4 Division of Hematology Oncology/Knight Cancer Institute/Oregon Health and Science University, Portland, OR 97239 USA; 5 Department of Public Health and Preventive Medicine/Oregon Health and Science University, Portland, OR 97239 USA; 6 Senior Scholar in the Center for Health Care Ethics/Oregon Health and Science University, Portland, OR 97239 USA

**Keywords:** Drug development, Cancer therapy

Although some cancer drugs offer large, indisputable benefits^1^, many drugs improve outcomes only marginally^2^. Recognizing the need to develop therapies of meaningful benefit to our patients, both the American Society of Clinical Oncology (ASCO)^3^ and the European Society of Medical Oncology (ESMO)^4^ have issued expert guidelines stating the magnitude of benefit that is clinically meaningful. These groups define clinically meaningful as whether drugs meet benchmarks of improvements in overall and progression free survival. For example, the ASCO guidelines propose that a new chemotherapeutic result in a relative increase in the median OS of at least 20% or 2.5–6 months^3^.

Prior groups have compared approved drugs^5^ and randomized trials^6^ against the ASCO and ESMO thresholds; however, to our knowledge, no analysis has compared the ASCO and ESMO thresholds against oncologist’s use of the phrase “meaningful benefit” in the published literature.

We sought biomedical articles where authors explicitly endorsed or stated that some numerical improvement in a clinical outcome seen in a randomized controlled trial constituted a meaningful benefit for a particular cancer indication.

We searched Google Scholar with the terms “meaningful benefit” and “oncology” or “meaningful benefit” and “cancer,” and limited our results to 2014 and 2015, as we were concerned with recent usage. Each article was reviewed by J.J.D. who identified the claim of meaningful benefit. Our study was conducted between November 2015 and March 2016.

Articles were excluded if: the article did not pertain to the field of oncology, the authors did not refer to a specific drug or combination, the authors were not claiming a meaningful benefit (i.e., they were saying a meaningful benefit does not exist), or the article did not reference a randomized trial and no such trial could be found.

We extracted changes in overall survival (OS), progression free survival, or other clinical endpoints between intervention and control arms that were deemed meaningful benefit. Descriptive statistics is provided. We compared author’s usage against the ESMO and ASCO guidelines.

We used the method of Kumar et al.^5^ for cancers not included in ASCO initial guidelines. Specifically, the group sought to adhere to the spirit of ASCO guidelines and gave credit for a PFS or OS of 2.5 months accompanied by a relative improvement of 25%.

We reviewed 559 articles in 2014 and 2015, and identified 53 claims of meaningful benefit in randomized studies. One was in the adjuvant setting (NSCLC), three were neoadjuvant (1 rectal, 2 urothelial), 49 were in the advanced or metastatic setting.

Of the 49 claims in the advanced/metastatic setting, 25 described median PFS improvement, 14 described median OS improvement, and 10 used another measure of benefit. These claims concerned 14 difference tumor types (Table 1).

**Table 1:** Cancer types where meaningful benefit was used

Cancer types reported in the advanced or metastatic setting	
Cancer type	Instances
Pancreatic	8
Breast	7
Non-small cell lung	7
Prostate	6
Colorectal	6
Myeloproliferative neoplasm	3
Melanoma	2
Thyroid	2
Glioblastoma	2
Ovarian	2
Gastric	1
Neuroendocrine	1
Acute myeloid leukemia	1
Germ-cell	1

**Fig. 1:**
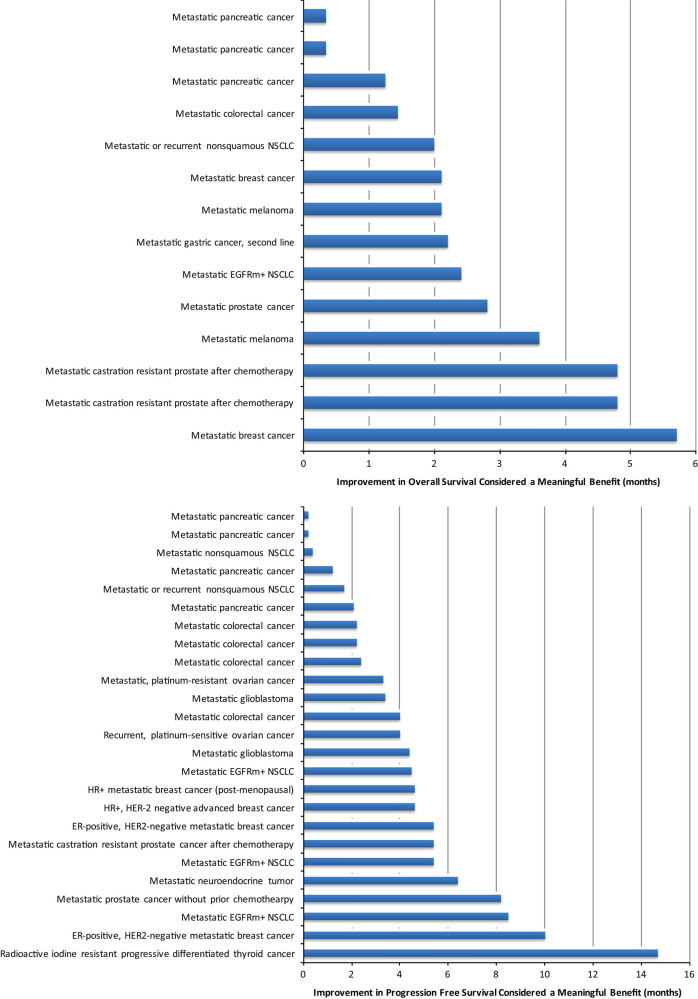
Top panel. Magnitude of improvement in median overall survival deemed a meaningful benefit. Bottom Panel. Magnitude of improvement progression free survival deemed a meaningful benefit

The median improvement in OS thought to constitute a meaningful benefit was 2.2 months (range 0.33–5.7 months). These are shown in Fig. 1 top panel. The median improvement in PFS thought to be meaningful was 4.0 month (range 0.2–14.7 months). These are shown in Fig. 1 bottom panel.

Among 14 claims of meaningful benefit based on median OS, 6 (43% 95% CI 18–71%) met ASCO and 4 (29% 95% CI 8–64%) met ESMO guidelines. Among 25 claims of meaningful benefit based on PFS, 17 (68% 95% CI 47–85%) met ASCO and 17 (68% 95% CI 47–85%) met ESMO guidelines.

Our results suggest that academic oncologists occasionally use the phrase “meaningful benefit” to describe a gain that does not meet expert, consensus guidelines. This happens 32% of the time for progression free survival and 57% and 71% of the time for overall survival, based on American and European standards, respectively. Given that the ASCO and ESMO thresholds are modest, we believe real world usage that falls short of this is setting the bar too low for our patients.

Future research should explore what magnitudes of benefit patients consider meaningful benefit, and whether these might serve as an externally valid metric for professional societies.
